# Anti-Thrombotic Effects of *Coprinus comatus* Fibrinolytic Enzyme in Zebrafish

**DOI:** 10.3390/nu17142358

**Published:** 2025-07-18

**Authors:** Yan Jing, Jinyu Wang, Yating He, Zedan Liu, Xiaolan Liu

**Affiliations:** 1 College of Food and Bioengineering, Qiqihar University, Qiqihar 161006, China; foodjingyan@126.com (Y.J.); 2024940516@qqhru.edu.cn (Y.H.); 2024940523@qqhru.edu.cn (Z.L.); 2 Key Laboratory of Corn Deep Processing Theory and Technology of Heilongjiang Province, Qiqihar 161006, China

**Keywords:** *Coprinus comatus*, fibrinolytic enzyme, thrombolysis and anticoagulation

## Abstract

Objectives: This study investigated the antithrombotic properties of a fibrinolytic enzyme (CFE) purified from the culture supernatant of *Coprinus comatus* using a zebrafish thrombosis model. Methods: A phenylhydrazine-induced thrombosis model was employed to evaluate the in vivo thrombolytic efficacy and mechanisms of CFE. Results: CFE significantly attenuated thrombogenesis by inhibiting erythrocyte aggregation in the caudal vessels, reducing staining intensity (3.61-fold decrease) and staining area (3.89-fold decrease). Concurrently, CFE enhanced cardiac hemodynamics, increasing erythrocyte staining intensity (9.29-fold) and staining area (5.55-fold) while achieving an 85.19% thrombosis inhibition rate. Behavioral analysis confirmed improved motility, with CFE-treated zebrafish exhibiting 2.23-fold increases in total movement distance and average speed, alongside a 3.59-fold extension in active movement duration. Mechanistically, ELISA revealed the multi-pathway activity of CFE, promoting fibrinolysis through reductions in plasminogen, fibrinogen, and D-dimer; inhibiting platelet activation via downregulation of prostaglandin-endoperoxide synthase (PTGS), thromboxane A2 (TXA2), P-selectin, and von Willebrand factor (vWF); and modulating coagulation cascades through elevated protein C and tissue factor pathway inhibitor (TFPI) with concurrent suppression of coagulation factor VII (FVII). Conclusions: These results indicate that the fibrinolytic enzyme CFE, derived from *Coprinus comatus*, exerts potent antithrombotic effects, supporting its potential as a basis for fungal-derived natural antithrombotic functional food ingredients.

## 1. Introduction

Globally, thrombus formation represents a leading cause of mortality and morbidity [1,2]. The core pathological mechanisms involve hyperactivation of the coagulation system, impaired fibrinolytic function, and abnormal platelet activation, ultimately leading to intravascular thrombosis [3,4,5]. Although thrombolytic agents, such as tissue plasminogen activator (t-PA) and urokinase-type plasminogen activator (u-PA), have been extensively employed to relieve thrombosis, they still present significant limitations, including an elevated risk of bleeding, a short plasma half-life, and a lack of target specificity [6,7]. Consequently, exploring novel fibrinolytic enzymes from natural sources that combine high efficacy and safety, and developing them into functional food ingredients with antithrombotic properties, holds significant potential for preventing thrombus formation. In recent years, macrofungi have attracted widespread attention due to their roles as both food and medicine. They are not only a rich source of nutrition but also a prolific producer of bioactive secondary metabolites, possessing antibacterial, immunomodulatory, antioxidant, and anti-tumor properties [8,9,10,11,12,13]. Among them, *Coprinus comatus*, a medicinal and edible fungus, has been documented to possess antioxidant, hypoglycemic, and immunomodulatory properties [14,15,16]. However, its capacity to produce fibrinolytic enzymes, along with its antithrombotic potential and the underlying mechanisms, remains to be systematically elucidated. Therefore, the exploration of naturally derived fibrinolytic enzymes from *Coprinus comatus* and their antithrombotic mechanisms would establish a scientific foundation for formulating functional food products with thromboembolism-preventive functions.

Fibrinolytic enzymes, a class of proteases that directly degrade fibrin or activate the fibrinolytic system, play a critical role in thrombolysis. Studies have demonstrated that fungal-derived fibrinolytic enzymes, such as those from *Pleurotus ferulae* [10], *Cordyceps militaris* [11], and *Agrocybe aegerita* [12], exhibit potent fibrinolytic and anticoagulant activities. The zebrafish (*Danio rerio*), owing to its high homology with humans in coagulation-fibrinolytic pathways, optical transparency of embryos for real-time observation, and suitability for high-throughput screening, has emerged as an ideal model organism for investigating dynamic thrombus formation and screening bioactive components with antithrombotic properties [17,18].

In this study, we systematically investigated the antagonistic effects of a highly purified, naturally derived *Coprinus comatus* fibrinolytic enzyme (CFE) on tail thrombosis in zebrafish. A multi-dimensional evaluation system was employed, incorporating microscopic observation of the zebrafish thrombosis model, analysis of erythrocyte aggregation, assessment of thrombosis inhibition, hemodynamic evaluation, and measurement of key thrombotic metabolic factors, comprehensively elucidating the antithrombotic activity of CFE in zebrafish. This research has identified novel natural sources of food-derived antithrombotic active factors and confirmed their in vivo biological efficacy against thrombosis. These findings not only provide a crucial theoretical foundation for the development of functional food ingredients but also establish a comprehensive methodology for evaluating antithrombotic activity.

## 2. Materials and Methods

### 2.1. Materials and Chemicals

Zebrafish were purchased from the National Zebrafish Resource Center. The animal study protocol was approved on 8 March 2023 by the Animal Ethics Committee of the College of Food and Bioengineering, Qiqihar University (Approval No. 2023-003). Phenylhydrazine (PHZ) was purchased from the Shanghai Shenggong Biological Engineering Co., Ltd. (Shanghai, China). O-Anisidine was purchased from Shanghai Guyan Technology Co., Ltd. (Shanghai, China). Dimethyl sulfoxide (DMSO) was purchased from Shanghai Guyan Technology Co., Ltd. (Shanghai, China). Phenylthiourea (PTU) was purchased from the Shanghai Shenggong Biological Engineering Co., Ltd. (Shanghai, China).

### 2.2. Zebrafish Breeding and Embryo Collection

The husbandry and breeding of zebrafish were conducted following the protocols outlined in The Zebrafish Book [19]. Zebrafish were maintained in a recirculating aquaculture system at a temperature of 28 ± 0.5 °C, pH of 6.5–7.5, and conductivity of 450–550 μS·cm^−1^, under a 14 h:10 h light-dark cycle. Healthy, sexually mature zebrafish were selected at a female-to-male ratio of 1:2 and placed in breeding tanks for paired spawning. Embryos were collected 1 h post-spawning and rinsed with system water. The collected healthy embryos were then cultured in embryo medium containing 200 μmol/L phenylthiourea (PTU) to inhibit melanin synthesis, facilitating subsequent observation of thrombus formation during modeling. After hatching, larvae were fed freshly hatched brine shrimp nauplii twice daily to support growth and development [20,21].

### 2.3. Zebrafish Thrombosis Modeling and Test Groups

Normally developed 6-day post-fertilization (6 dpf) zebrafish larvae were screened using a stereomicroscope and randomly allocated into a blank control group (treated with embryo culture water) and a thrombus model group (treated with phenylhydrazine, PHZ), with 30 larvae per well in a 6-well plate for the experiment. The thrombus model group was treated with PHZ at gradient concentrations of 1, 1.5, 2, 5, 10, and 15 μM, followed by co-incubation in a 28 °C constant temperature incubator for 16 h [21,22]. The thrombus formation characteristics in the caudal vein were observed using a stereomicroscopic imaging system. The thrombus formation rate and mortality rate of each group were systematically recorded, and the staining intensity of the thrombus area in the caudal vein was quantitatively analyzed using Image-Pro Plus 6 software to establish the dose–effect relationship with PHZ concentration. The experiment was independently replicated three times to ascertain the optimal concentration of PHZ for thrombosis modeling. Normally developed 6-day post-fertilization (6-dpf) juvenile zebrafish were randomly allocated into five groups: a blank control group, a PHZ model group, a urokinase positive control group, and low-, medium-, and high-dose CFE intervention groups (*n* = 30/group). The positive control group and the CFE intervention group were first pre-incubated for 12 h, followed by incubation with 2 μM PHZ for an additional 16 h. The detailed treatment protocols for each group are presented in Table 1. The experiment was conducted under constant 28 °C incubation conditions. The data were analyzed using SPSS 25.0 for variance analysis.

$$\mathrm{Thrombus}\;\mathrm{formation}\;\mathrm{rate}\%=\frac{\mathrm{Number}\;\mathrm{of}\;\mathrm{thrombus}-\mathrm{positive}\;\mathrm{larvae}}{\mathrm{Total}\;\mathrm{number}\;\mathrm{of}\;\mathrm{surviving}\;\mathrm{larvae}}\times 100\%$$

### 2.4. Effects of CFE on the Area and Intensity of Erythrocyte Staining in Zebrafish Heart and Ishtail

The zebrafish thrombus model was established as described in Section 2.3. Twenty normally developing juvenile zebrafish were randomly selected from each group for o-diphenylamine staining [23]. During the staining procedure, the solution in the microplate was first discarded and then replaced with an equal volume of staining solution. After incubation at 28 °C for 20 min, the samples were washed three times and subsequently fixed with 4% paraformaldehyde. The aggregation of erythrocytes in the zebrafish’s heart and tail regions was observed and documented with photography. The Image-Pro Plus 6 software was utilized to quantify the stained area (*S*) and staining intensity of erythrocytes in both the heart and tail regions, enabling the calculation of the thrombus inhibition rate.

$$\mathrm{Thrombus}\;\mathrm{inhibition}\;\mathrm{rate}\%=\frac{S\;(\mathrm{Model}\;\mathrm{group})-S\;(\mathrm{Experimental}\;\mathrm{group})}{S\;(\mathrm{Model}\;\mathrm{group})}\times 100\%$$

### 2.5. Behavioral Analysis of Zebrafish

The Zebralab 3.3 Viewpoint behavior recorder was utilized to conduct behavioral analysis by transferring zebrafish from different groups into 96-well plates, with one fish per well. Prior to the experiment, juvenile zebrafish were acclimated to a dark environment (28 °C) for 30 min. The experimental setup included alternating light and dark conditions every 30 min. Parameter settings were as follows: movement speeds less than 1 mm/s were classified as inactive or stationary; speeds between 1 and 3 mm/s were categorized as moderate activity; speeds greater than 3 mm/s indicated high activity, with a detection threshold of 20. The test control protocol specified that each cycle would commence 10 min after the start of the experiment, with a day–night ratio of 10 h:14 h [24,25]. After the data were collected, trajectory smoothing was automatically performed using EthoVision XT 15 software to analyze the impact of CFE on the zebrafish’s total moving distance, average speed, and the proportion of time spent moving.

### 2.6. Fibrinolysis Promotion of CFE In Vivo

Zebrafish from experimental groups (triplicates per group) were processed by washing with distilled water, removing residual liquid, and resuspending in 50 μL PBS (pH 7.4). The fibrinolysis markers plasminogen, fibrinogen, and D-dimer were quantified using ELISA.

### 2.7. AntiPlatelet Activation of CFE In Vivo

Zebrafish were grouped and subjected to thrombus modeling as detailed in Section 2.3, with triplicate samples per group. Following collection, samples were washed twice with distilled water, residual liquid was removed, and tissues were resuspended in 50 μL PBS (pH 7.4). Antiplatelet activity was assessed by using ELISA to quantify key platelet activation markers, including PTGS, TXA2, P-selectin, and vWF.

### 2.8. Anticoagulant Effect of CFE In Vivo

Using the established thrombus model (triplicates per group), zebrafish samples were washed with distilled water. Residual liquid was then removed, and the samples were then homogenized in 50 μL PBS (pH 7.4). Anticoagulant activity was evaluated by measuring regulators—protein C, TFPI, and FVII—using ELISA.

### 2.9. Statistical Analysis

All data were presented as the mean ± standard deviation and analyzed using SPSS Statistics 26 (SPSS Inc., Chicago, IL, USA). The Duncan test was employed to evaluate the significance of differences, with superscript letters denoting significant differences at *p* < 0.05.

## 3. Results and Discussion

### 3.1. Determination of Maximum Tolerance Concentration of CFE

**Figure 1:**
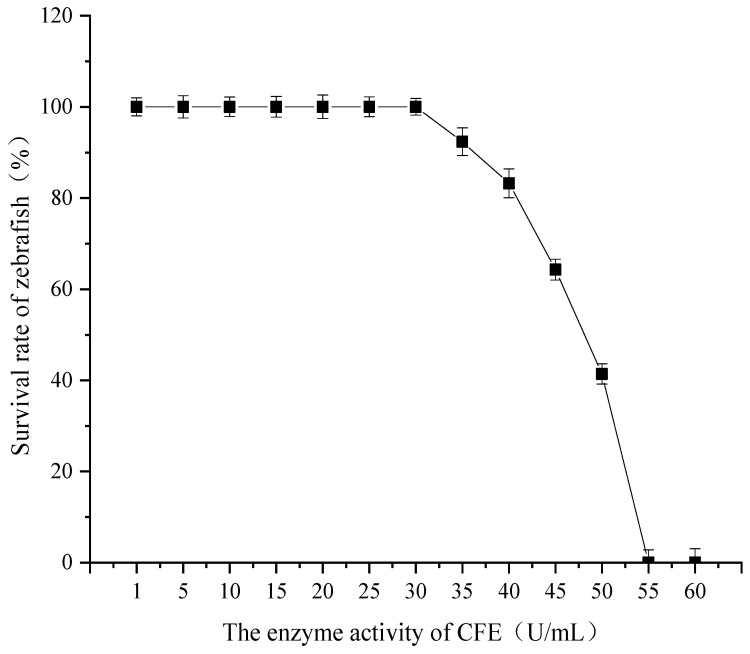
Determination of the maximum tolerated concentration of CFE.

The maximum tolerated dose of CFE in zebrafish was investigated, and the results are illustrated in Figure 1. When the CFE enzyme activity ranged from 1 U/mL to 30 U/mL, the survival rate of juvenile zebrafish remained consistent at 100%. However, as the enzyme activity exceeded 30 U/mL, a significant decreasing trend in the survival rate was observed. Specifically, at an enzyme activity level of 35 U/mL, the survival rate decreased to 92.34%, and it continued to decline progressively with increasing enzyme activity. At 55 U/mL, the survival rate of zebrafish reached 0%. These findings suggest that CFE enzyme activities below 30 U/mL do not induce mortality in zebrafish, indicating no apparent toxicity within this range. Therefore, the maximum tolerance concentration of CFE for zebrafish was determined to be 30 U/mL. Subsequently, the enzyme activities for the low, medium, and high CFE test groups were established at 10 U/mL, 20 U/mL, and 30 U/mL, respectively.

### 3.2. Determination of Phenylhydrazine Concentration for Optimal Thrombus Modeling

**Figure 2:**
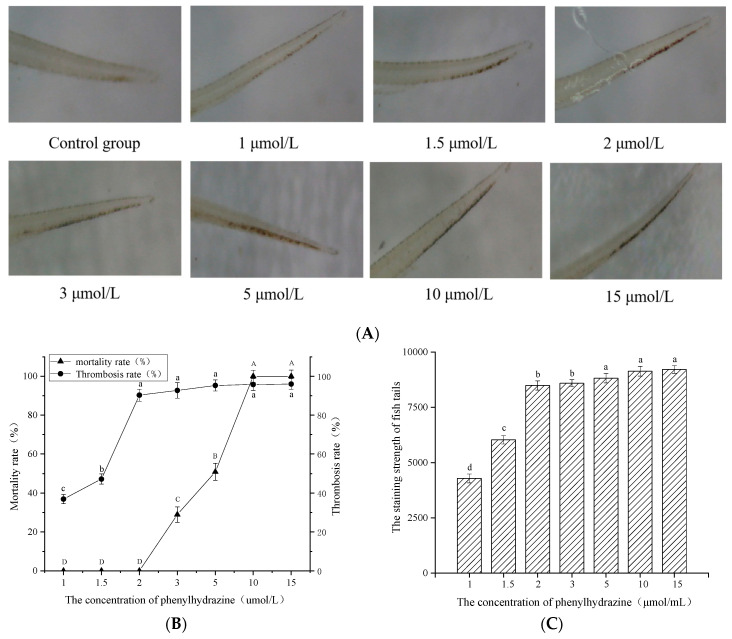
Construction of zebrafish phenylhydrazine-induced thrombus model. (**A**) Fishtail microscopic observation of zebrafish phenylhydrazine-induced thrombus model; (**B**) Relationship between zebrafish thrombosis rate and mortality; (**C**) Fishtail erythrocyte staining intensity. Different uppercase letters (A, B, C, D) and lowercase letters (a, b, c, d) indicated statistically significant differences between groups (*p* < 0.05) as determined by multiple comparison tests.

To investigate the effect of different concentrations of PHZ on the thrombosis rate in zebrafish, a composite curve of PHZ concentration versus zebrafish mortality and thrombosis rate was plotted. This was combined with the degree of erythrocyte aggregation and staining intensity in the caudal veins of zebrafish to determine the optimal PHZ concentration for thrombosis modeling. The results, as shown in Figure 2, indicate that, compared to the blank control group, PHZ concentrations ranging from 1 to 15 μmol/L could induce thrombosis in the caudal veins of zebrafish. Both the thrombosis rate and the staining intensity of the caudal vein exhibited a dose-dependent relationship with PHZ concentration. Microscopic observations revealed that at PHZ concentrations of 1–1.5 μmol/L, erythrocyte aggregation occurred in the caudal vein, with a zebrafish mortality rate of 0%, although the thrombosis rate was relatively low. When the PHZ concentration was in the range of 2–5 μmol/L, the thrombosis rate in the caudal vein increased with rising PHZ concentration. At a PHZ concentration of 2 μmol/L, the zebrafish mortality rate was 0%, while the thrombosis rate reached 90.28%. When the PHZ concentration exceeded 2 μmol/L, zebrafish mortality began to occur. At PHZ concentrations of 5–15 μmol/L, the thrombosis rate in the caudal vein exceeded 95% and stabilized, but the zebrafish mortality rate was significantly higher. At PHZ concentrations greater than 10 μmol/L, the mortality rate reached 100%. Therefore, a PHZ concentration of 2 μmol/L was determined to be the optimal concentration for establishing a stable in vivo zebrafish caudal vein thrombosis model.

### 3.3. Microscopic Evaluation of Erythrocyte Aggregation

Microscopic observation was performed to evaluate the aggregation of erythrocytes in the hearts and caudal veins of zebrafish across different experimental groups, thereby assessing the effect of CFE on erythrocytes in zebrafish. During the diastolic phase of the heart, arterial pressure is lower than venous and tissue pressure, which facilitates the flow of blood from the veins toward the heart. However, once a thrombus forms in vivo, the slower venous blood flow promotes the aggregation of erythrocytes at the thrombus site, impeding blood return and leading to a reduction in erythrocyte count in the heart.

Consequently, the degree of erythrocyte aggregation and staining intensity in the caudal veins of zebrafish is positively correlated with thrombus formation, whereas the degree of erythrocyte aggregation and staining intensity in the heart is inversely correlated with thrombus formation [20].

**Figure 3:**
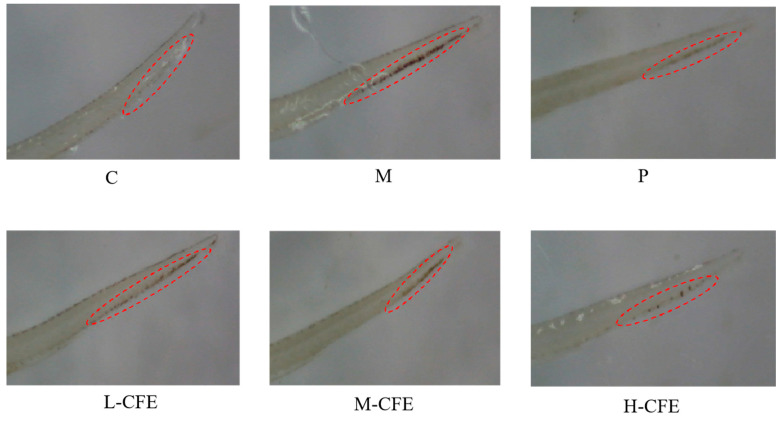
Microscopic observation of the zebrafish tails for each experimental group. C represents the Control group; M represents the Thrombus model group; P represents the Positive control group; L-CFE represents the Low-dose CFE group; M-CFE represents the Medium-dose CFE group; H-CFE represents the High-dose CFE group. The red circles highlight the differential erythrocyte aggregation patterns observed microscopically in the caudal region of zebrafish across the experimental groups.

**Figure 4:**
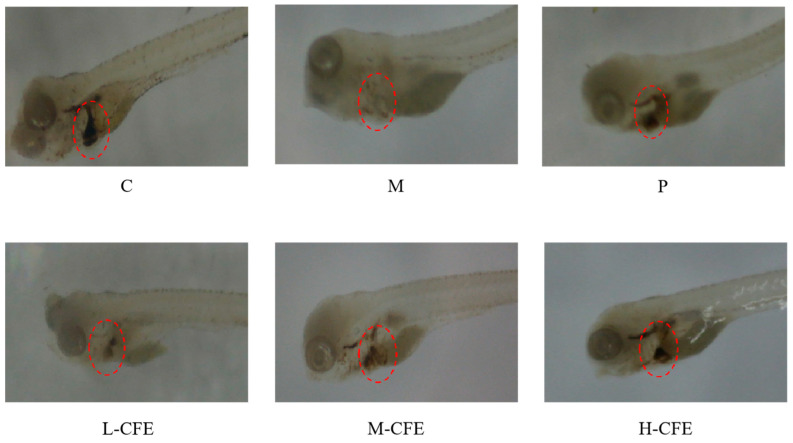
Microscopic observation of the zebrafish hearts from each experimental group. C represents the Control group; M represents the Thrombus model group; P represents the Positive control group; L-CFE represents the Low-dose CFE group; M-CFE represents the Medium-dose CFE group; and H-CFE represents the High-dose CFE group. The red circles highlight the differential erythrocyte aggregation patterns observed microscopically in the hearts of zebrafish across experimental groups.

The microscopic observations of erythrocyte aggregation in the caudal veins and hearts of zebrafish are presented in Figure 3 and Figure 4, respectively. Compared to the blank control group, the model group displayed a markedly decreased distribution of erythrocytes in the heart and exhibited significant thrombus formation in the caudal vein. In contrast, the positive control group and the high-, medium-, and low-dose CFE groups all showed varying degrees of alleviation in caudal vein thrombus formation, as well as an enhanced distribution of erythrocytes in the heart. Notably, the high-dose CFE group demonstrated an erythrocyte distribution pattern in zebrafish that closely resembled that of the blank control group. These findings suggested that CFE could effectively inhibit erythrocyte aggregation in the caudal veins of zebrafish and promote efficient blood return to the heart.

**Figure 5:**
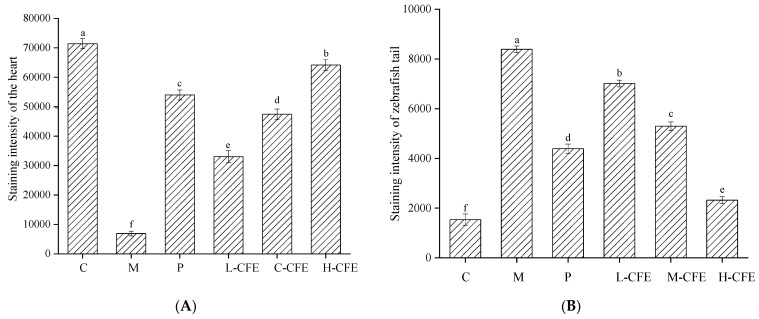
Analysis of staining intensity of zebrafish hearts and tails. (**A**) The staining intensity of zebrafish hearts in different experimental groups. (**B**) The staining intensity of zebrafish tails in different experimental groups. C represents the Control group; M represents the Thrombus model group; P represents the Positive control group; L-CFE represents the Low-dose CFE group; M-CFE represents the Medium-dose CFE group; H-CFE represents the High-dose CFE group. Different lowercase letters indicated statistically significant differences between groups (*p* < 0.05) as determined by multiple comparison tests.

**Figure 6:**
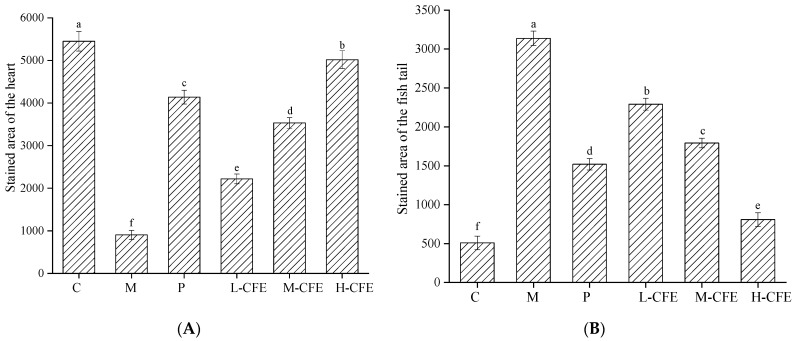
Analysis of heart and tail stained areas of zebrafish. (**A**) The stained areas of zebrafish hearts in different experimental groups. (**B**) The stained areas of zebrafish tails in different experimental groups. C represents the Control group; M represents the Thrombus model group; P represents the Positive control group; L-CFE represents the Low-dose CFE group; M-CFE represents the Medium-dose CFE group; H-CFE represents the High-dose CFE group. Different lowercase letters indicated statistically significant differences between groups (*p* < 0.05) as determined by multiple comparison tests.

Figure 5A,B and Figure 6A,B illustrate the effects of CFE on the intensity and area of red blood cell staining in the hearts and tail regions of zebrafish, respectively. As shown in Figure 5A and Figure 6A, following thrombus modeling, the staining intensity and staining area in the hearts of the model group decreased compared to the blank control group. This indicated that under PHZ induction, blood could not properly return to the cardiac arteries, leading to obstructed blood flow and the formation of thrombi within the zebrafish. In contrast, the positive control group and the low-, medium-, and high-dose CFE groups exhibited increased staining intensity and staining area of erythrocytes in the heart compared to the model group. This suggested that CFE intervention inhibited PHZ-induced blood coagulation, promoted blood return to the heart, and consequently increased the staining intensity and staining area of erythrocytes in the heart, thereby restoring normal blood flow conditions in the zebrafish.

The effects of CFE on the staining intensity and staining area of erythrocytes in the caudal of zebrafish were illustrated in Figure 5B and Figure 6B. Compared with the blank control group, the staining intensity and staining area of erythrocytes in the tail veins of zebrafish in the model group were markedly enhanced, indicating that PHZ induces thrombosis in these veins, leading to the accumulation of erythrocytes. Conversely, the positive control group and the low-, medium-, and high-dose CFE groups demonstrated a significant decrease in both the staining intensity and the stained area of erythrocytes in the fishtail compared to the model group. This suggested that CFE intervention successfully suppresses PHZ-induced caudal vein thrombosis or resolves the formed microthrombi, thereby restoring normal vascular blood flow. The findings of this study demonstrate a high degree of consistency with previous research on zebrafish thrombosis models. In a study conducted by Yang et al. [21], a PHZ-induced zebrafish model was employed to investigate the antithrombotic effects of Lasianthus. Their results indicated that thrombosis formation was associated with reduced red blood cell staining intensity in the heart and increased thrombosis in the tail vein, which aligns closely with the observations from the PHZ model group in the present study. Furthermore, the pathological characteristics, such as cardiac venous congestion and diminished blood flow, identified by Zhou et al. [23] during their investigation into the cardiovascular toxicity of edebenone, provide additional support for the presence of thrombosis-induced microcirculatory disturbances.

### 3.4. The Rate of Thrombus Inhibition In Vivo

**Figure 7:**
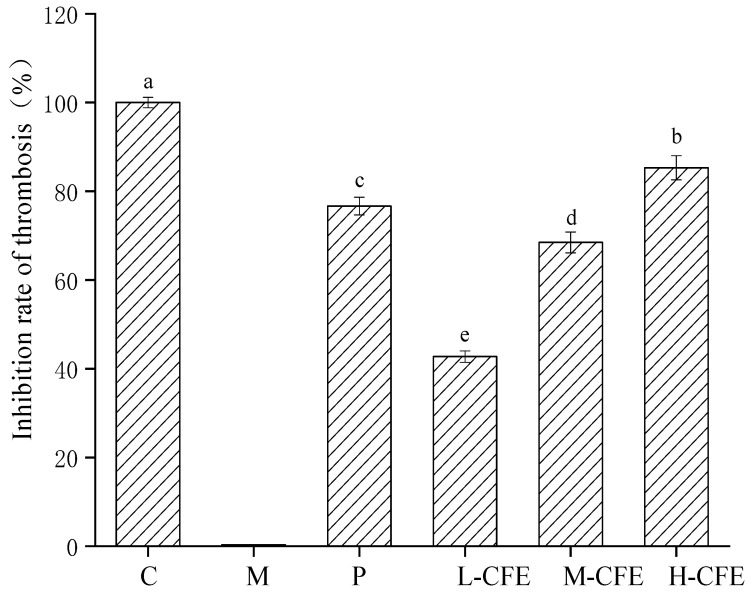
Effects of CFE on thrombus inhibition rate in zebrafish. C represents the Control group; M represents the Thrombus model group; P represents the Positive control group; L-CFE represents the Low-dose CFE group; M-CFE represents the Medium-dose CFE group; H-CFE represents the High-dose CFE group. Different lowercase letters indicate statistically significant differences between groups (*p* < 0.05) as determined by multiple comparison tests.

We calculated the inhibition rate of CFE on zebrafish tail thrombosis by analyzing the staining intensity and staining area of zebrafish erythrocytes before and after thrombosis modeling, as presented in Figure 7. Compared with the thrombus model group, the urokinase positive control group and the low-, medium-, and high-dose CFE groups had significant inhibitory effects on thrombus formation in zebrafish, with inhibitory rates of 76.66%, 42.73%, 68.48%, and 85.19%, respectively. The results showed that CFE could inhibit fishtail thrombosis induced by PHZ. As CFE enzyme activity increased, its inhibitory effect on thrombosis exhibited a graded enhancement. Specifically, the high-dose CFE group demonstrated a significantly stronger thrombus inhibitory effect compared to the urokinase-positive control group at the same dosage, followed by the medium-dose group. While the low-dose group exhibited some inhibitory activity, the effect was comparatively weaker. This dose–response relationship (high dose > positive control > medium dose > low dose) confirmed that the inhibitory effect of CFE on tail thrombosis in zebrafish was dose-dependent and that CFE’s antithrombotic activity is positively correlated with its enzymatic activity. It was hypothesized that CFE might exert a certain protective effect against cell damage induced by PHZ, or it could dissolve small thrombi formed in zebrafish, thereby preventing vascular obstruction and inhibiting clot formation.

### 3.5. Effect of CFE on Zebrafish Behavior

The behavioral analysis of zebrafish typically encompasses studies on locomotor behavior, social behavior, feeding behavior, and stress responses, which can reflect the physiological and psychological states of the fish. Locomotor behavior analysis is one of the most fundamental and widely utilized approaches, including free swimming, open-field tests, and light–dark avoidance tests, for which swimming activity parameters are regarded as critical indicators for assessing neural function and overall health. Zebrafish behavioral research typically employs behavioral tracking systems for real-time dynamic monitoring, enabling the detection of the movement trajectories and activity levels of zebrafish larvae under both dark and light stimulation [25,26,27]. In this experiment, the total distance moved, average movement speed, and movement time proportion were tracked and measured during free swimming to investigate the effects of CFE on zebrafish behavior.

The effect of CFE on the total distance moved by zebrafish is presented in Table 2. The total distance moved by the blank control group was 4244.07 ± 182.72 mm, whereas that of the thrombus model group was significantly reduced at 1117.90 ± 195.30 mm. Both the positive control group and the high-, medium-, and low-dose CFE groups demonstrated an increase in the total distance moved by the zebrafish. Specifically, the positive control group exhibited a recovery in movement distance to 2877.69 ± 208.27 mm, while the high-dose CFE group showed a more pronounced recovery to 3615.78 ± 189.54 mm, approaching the level of the blank control group. These findings suggested that CFE intervention inhibited PHZ-induced thrombus formation in the tails of zebrafish, thereby improving systemic blood circulation. Enhanced blood flow was conducive to maintaining the normal function of the neuromuscular system, leading to the restoration of locomotor activity in the zebrafish.

**Figure 8:**
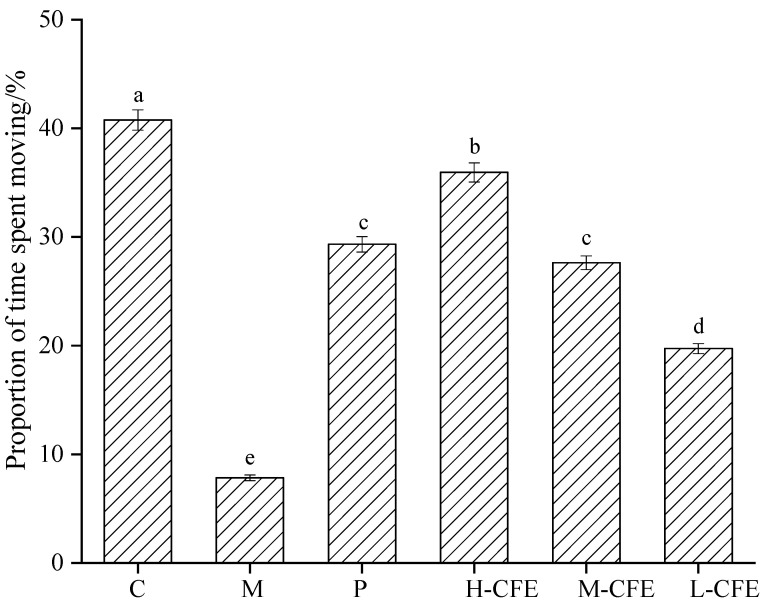
Effects of CFE on the proportion of time spent moving by zebrafish. C represents the Control group; M represents the Thrombus model group; P represents the Positive control group; L-CFE represents the Low-dose CFE group; M-CFE represents the Medium-dose CFE group; H-CFE represents the High-dose CFE group. Different lowercase letters indicated statistically significant differences between groups (*p* < 0.05) as determined by multiple comparison tests.

The effects of CFE on the average movement speed of the zebrafish are presented in Table 3. Compared to the blank control group, the average movement speed of zebrafish in the PHZ-induced thrombus model group decreased from 14.20 ± 0.65 mm/s to 3.73 ± 0.42 mm/s. However, the positive control group and the high-, medium-, and low-dose groups all exhibited varying degrees of recovery in the average movement speed of zebrafish. Specifically, the high-dose CFE group restored the average movement speed to 12.06 ± 0.37 mm/s, which was superior to that of the positive control group and approached the level of the blank control group. As illustrated in Figure 8, following the establishment of PHZ thrombosis modeling, the ratio of zebrafish movement time to total observation time decreased. This suggests that thrombus formation resulted in reduced mobility in the zebrafish. Both the positive control group and the high-, medium-, and low-dose groups of CFE showed an increase in the proportion of movement time. Notably, the medium-dose CFE group showed no significant difference in the proportion of movement time compared to the positive control group, while the effect of the high-dose CFE group was closer to that of the blank control group. The results of the behavioral study demonstrated that CFE enhanced the motor abilities of the zebrafish. Under CFE intervention, the total distance moved and average movement speed of the zebrafish increased, indicating improved activity and movement frequency during the experiment. The increase in the proportion of movement time suggested that CFE enabled zebrafish to maintain an active state over a prolonged period, reflecting an improvement in physiological condition. From the behavioral study of the zebrafish, it was evident that CFE could enhance their activity level and behavioral liveliness, indicating potential biological effects that improve blood circulation and enhance motor ability. The findings of Wang et. al. [28] were consistent with our experimental results, demonstrating that significant improvements in zebrafish locomotor parameters suggest effective inhibition of thrombus formation, which aligns with our conclusions.

### 3.6. Profibrinolytic Effect of CFE In Vivo

The core of the fibrinolytic system lies in the conversion of plasminogen to plasmin under the action of plasminogen activators, followed by the degradation of fibrin. This process involves zymogen activation, feedback amplification of enzymatic activity, and inhibition, ultimately achieving a dynamic equilibrium [29]. Plasminogen is synthesized by liver cells and subsequently distributed throughout the body via the bloodstream [30]. Fibrinogen, the most abundant clotting factor in plasma, undergoes cross-linking reactions mediated by thrombin, FXIIIa, and Ca^2+^ to form stable thrombi [31,32]. As a specific marker of fibrin degradation, D-dimer objectively reflects fibrinolytic activity levels [33,34]. This section focuses on investigating the effects of CFE on plasminogen, fibrinogen, and D-dimer.

**Figure 9:**
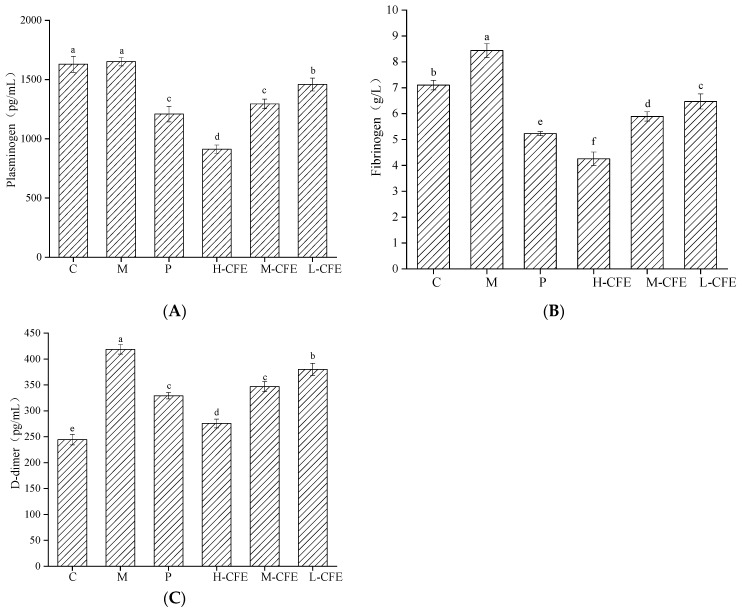
Fibrinolysis-promoting effect of CFE on zebrafish. (**A**) Concentration of plasminogen; (**B**) Concentration of fibrinogen; (**C**) Concentration of D-dimer. C represents the Control group; M represents the Thrombus model group; P represents the Positive control group; L-CFE represents the Low-dose CFE group; M-CFE represents the Medium-dose CFE group; H-CFE represents the High-dose CFE group. Different lowercase letters indicate statistically significant differences between groups (*p* < 0.05) as determined by multiple comparison tests.

The effects of CFE on fibrinolytic-related active factors in zebrafish are illustrated in Figure 9. Compared with the blank control group, the thrombotic model group exhibited a non-significant increasing trend in plasminogen levels but significant elevations in both fibrinogen and D-dimer contents (*p* < 0.05) in the zebrafish, suggesting that PHZ-induced inflammatory responses triggered pathological thrombus formation and subsequently activated compensatory fibrinolysis through the coagulation–fibrinolysis equilibrium mechanism. Both the positive control group and CFE at different doses demonstrated dose-dependent reductions in plasminogen, fibrinogen, and D-dimer levels in zebrafish. In our previous study [35], we demonstrated that CFE possessed plasminogen kinase activity. We therefore hypothesize that CFE could directly catalyze the conversion of plasminogen to plasmin through this enzymatic activity, while simultaneously compromising fibrinogen structural integrity. This proposed dual mechanism would consequently suppress fibrin polymer formation and decrease levels of its specific degradation product, D-dimer. This finding was consistent with the research conducted by Zheng et al. [36] on D-dimer as a marker of intravascular coagulation, which indicated that elevated D-dimer levels are closely associated with coagulation activation. The results suggest that CFE, by modulating the levels of plasminogen, fibrinogen, and D-dimer in the zebrafish, decreased the substrates for thrombus formation, enhanced the direct degradation of fibrin, activated the fibrinolytic system, and promoted fibrin degradation.

### 3.7. Antiplatelet Activation Effect of CFE In Vivo

Under physiological conditions, platelets remain in a resting state. Vascular endothelial injury activates the coagulation cascade, initiating the extrinsic pathway via tissue factor (TF) and factor VII (FVII), ultimately leading to the generation of activated factor X (FXa) and thrombin, which converts fibrinogen to fibrin. Subsequently, platelets undergo morphological changes via transmembrane signal transduction systems, initiating adhesion, aggregation, and release reactions that synergize with fibrin to form the thrombus core. This indicates that inhibiting platelet activation is an effective strategy for modulating thrombus formation [37,38]. PTGS, the rate-limiting enzyme in prostaglandin synthesis, catalyzes the conversion of arachidonic acid to prostaglandin G2 (PGG2), which is further reduced to prostaglandin H2 (PGH2) [39]. As a biosynthetic precursor of prothrombotic mediators such as TXA2, PGH2 significantly enhances platelet activation and aggregation risk. TXA2, generated via PTGS and thromboxane synthase pathways, exhibits potent vasoconstrictive and platelet-aggregating effects. However, due to its short half-life (~30 s), it rapidly metabolizes into the stable thromboxane B2 (TXB2), making TXB2 a common surrogate marker for TXA2 production in clinical assessments [40]. P-selectin, a platelet- and endothelial cell-specific adhesion molecule (140 kDa), is rapidly expressed on the membrane upon stimulation by thrombin and other agonists. It mediates leukocyte rolling and enhances platelet adhesion, serving not only as a cellular activation marker but also as a key inducer of thrombosis [41,42]. Additionally, vWF, encoded on the short arm of chromosome 12, drives initial thrombus formation by mediating platelet-collagen adhesion upon vascular injury [43,44]. Therefore, we investigated the effects of CFE on platelet activation-related mediators—PTGS, TXA2, P-selectin, and vWF—in zebrafish.

**Figure 10:**
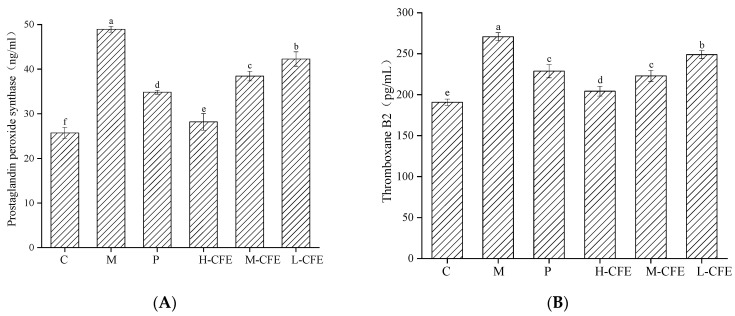

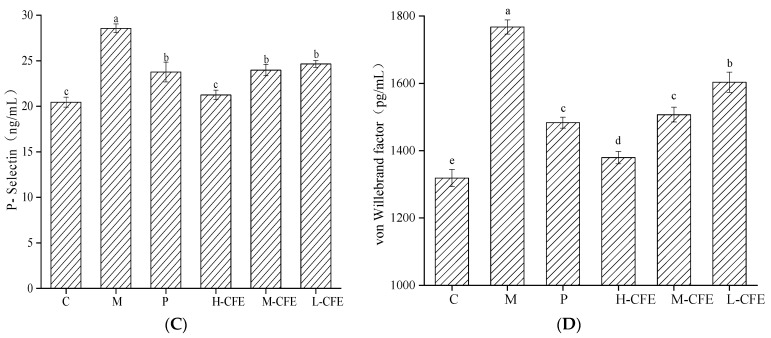
Anti-platelet activation of CFE in zebrafish. (**A**) Concentration of prostaglandin-endoperoxide synthase; (**B**) Concentration of thromboxane B2; (**C**) Concentration of P-selectin; (**D**) Concentration of von Willebrand factor. C represents the Control group; M represents the Thrombus model group; P represents the Positive control group; L-CFE represents the Low-dose CFE group; M-CFE represents the Medium-dose CFE group; H-CFE represents the High-dose CFE group. Different lowercase letters indicated statistically significant differences between groups (*p* < 0.05) as determined by multiple comparison tests.

The effects of CFE on platelet activation-related active factors are shown in Figure 10. Compared with the blank group, the thrombus model group exhibited significantly elevated levels of PTGS, TXA2, P-selectin, and vWF (*p* < 0.05), indicating that PHZ enhanced platelet activity and induced a hypercoagulable state by promoting arachidonic acid metabolism, increasing prostaglandin and thromboxane synthesis, and stimulating vWF secretion. Meanwhile, due to the onset of inflammation in the body, platelet activation was enhanced, leading to the rapid translocation of P-selectin to the cell surface. P-selectin binds to P-selectin glycoprotein ligand-1 (PSGL-1) on leukocytes, mediating their migration to the inflammatory site. This process enhanced the adhesion between platelets and leukocytes, platelets and platelets, as well as platelets and endothelial cells, thereby promoting the formation of stable thrombi [44]. Both the positive control and CFE-treated groups showed dose-dependent reductions in thrombotic markers, with the high-dose CFE achieving effects comparable to those of the blank control. The antithrombotic mechanism likely involved PTGS activity inhibition, leading to decreased PGI2 synthesis, which subsequently reduced TXB2 levels and P-selectin expression on platelets, attenuated vWF-mediated platelet-vessel wall adhesion, and improved inflammatory responses and blood flow [42,43,44]. These findings align with those of Wang et al. [28], who reported PTGS regulation in zebrafish thrombosis. Liu et al. [45] demonstrated TXA2-prostacyclin imbalance in thrombogenesis, and Kamil et al. [46] showed TXA2’s role in platelet activation via the ROS–TXA2 pathway. Sun et al. [47] found that P-selectin was an important cell adhesion factor and played an important role in regulating the blood system. Collectively, these findings suggest that CFE exerts an inhibitory effect on tail thrombosis in zebrafish by suppressing platelet activity.

### 3.8. Anticoagulation Effect of CFE In Vivo

Anticoagulation is a critical mechanism for inhibiting thrombus formation and maintaining blood fluidity. Protein C, a central anticoagulant component, regulates the coagulation cascade through its activated form by inactivating key clotting factors (FVIIIa, FVa, FIXa, and FXa) [48,49]. TFPI suppresses thrombin activation and prevents coagulation initiation by blocking the tissue factor (TF)-mediated coagulation pathway [50]. FVII, a liver-derived vitamin K-dependent glycoprotein, plays a pivotal role in the extrinsic coagulation pathway. Upon vascular injury, exposed TF binds FVII to form the TF-FVIIa complex, which subsequently activates FX and FIX, triggering the coagulation cascade and ultimately leading to fibrin clot formation [49,50]. This section investigated the effects of CFE on coagulation-related factors, including protein C, TFPI, and FVII, in zebrafish, and evaluated its anticoagulant properties.

**Figure 11:**
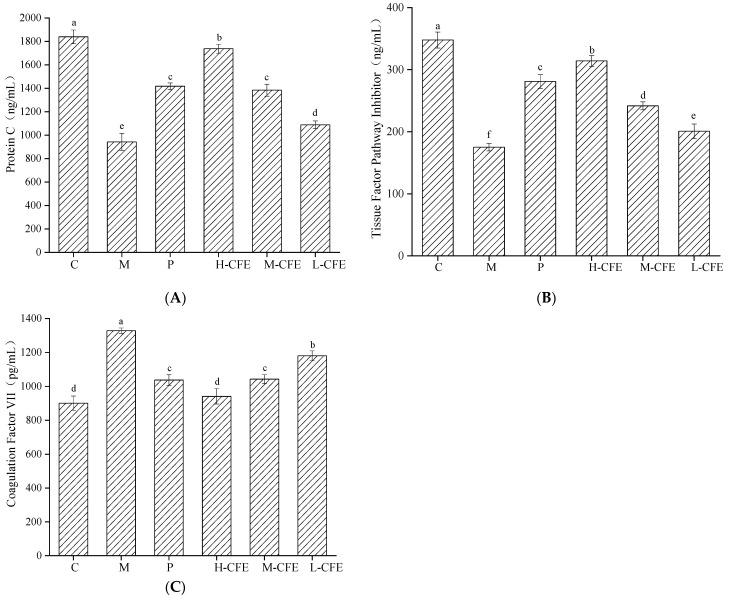
Anticoagulation effect of CFE on zebrafish. (**A**) Concentration of protein C; (**B**) Concentration of tissue factor pathway inhibitor; (**C**) Concentration of coagulation factor VII. C represents the Control group; M represents the Thrombus model group; P represents the Positive control group; L-CFE represents the Low-dose CFE group; M-CFE represents the Medium-dose CFE group; H-CFE represents the High-dose CFE group. Different lowercase letters indicate statistically significant differences between groups (*p* < 0.05) as determined by multiple comparison tests.

The effects of CFE on coagulation-related factors in zebrafish are illustrated in Figure 11. The experimental results demonstrated that protein C and TFPI levels in the thrombus model group of zebrafish were significantly decreased compared with the control group (*p* < 0.05), while FVII levels were markedly elevated. Mechanistic analysis revealed that phenylhydrazine (PHZ)-induced cellular damage triggered inflammatory responses, activating the coagulation system to promote thrombin generation, thereby suppressing protein C synthesis or accelerating its degradation. Concurrently, tissue factor (TF) exposure and subsequent binding with FVIIa activated the extrinsic coagulation pathway, leading to increased TFPI consumption. Compared with the model group, both urokinase and CFE treatment groups (particularly the high-dose CFE group) significantly reversed these alterations, with the high-dose CFE group exhibiting effects approaching the levels observed in the control group. CFE exerted anticoagulant effects through dual mechanisms: on the one hand, it reduced protein C consumption by facilitating the formation of protein *C*-protein S complexes to inhibit factor VIII (FVIII) and factor V (FV) activities. On the other hand, it upregulated TFPI expression, thereby blocking the coagulation cascade via TFPI-TF complex formation and suppressing FVII activity. Given the pivotal role of FVII in initiating the extrinsic coagulation pathway, its downregulation substantially impeded coagulation processes. This study confirmed that CFE synergistically modulates the expression levels of protein C, TFPI, and FVII, effectively enhancing anticoagulant activity while inhibiting coagulation cascades, ultimately achieving antithrombotic effects. Liu et al. investigated the function of protein C in a mouse model through genotypic and phenotypic analyses, revealing that changes in protein C levels can modulate coagulation-related indicators and influence thrombus formation [51]. Raman Revathi et al. [52] demonstrated through zebrafish experiments that TFPI functions as an anticoagulant by inhibiting factors VIIa and Xa in the coagulation cascade, thereby suppressing blood clotting.

## 4. Conclusions

This study confirmed that CFE exhibits significant antithrombotic efficacy in a zebrafish thrombosis model. CFE effectively ameliorated thrombosis-induced erythrocyte aggregation, caudal circulation impairment, and cardiac dysfunction, while dose-dependently attenuating thrombus formation in a manner closely correlated with its enzymatic activity. Furthermore, CFE significantly alleviated thrombosis-induced behavioral locomotor dysfunction. Mechanistic investigations revealed that CFE exerted its antithrombotic effect through synergistic multi-target actions: inhibiting platelet activation, as evidenced by reduced levels of key activation factors TXA2, PTGS, P-selectin, and vWF; impeding the coagulation cascade, as evidenced by elevated levels of anticoagulant factors protein C and TFPI alongside decreased levels of procoagulant factor FVII; and promoting fibrinolysis, as evidenced by reduced levels of plasminogen, D-dimer, and fibrinogen. In summary, by synergistically regulating key targets involved in platelet activation, the coagulation system, and the fibrinolytic system, CFE effectively inhibits caudal vein thrombus formation and ameliorates associated pathophysiological damage in zebrafish. These findings not only elucidate the in vivo antithrombotic mechanism of CFE but also provide a crucial theoretical foundation and potential applications for its use as a functional food ingredient in the prevention and management of thrombosis.

## Figures and Tables

**Table 1:** Experimental protocol.

Groups	Experimental Treatment Method
Control group (C)	Embryo culture water
Thrombus model group (M)	2 μmol/L PHZ + Embryo culture water
Positive control group (P)	30 U/mL urokinase + 2 μmol/L PHZ + Embryo culture water
Low-dose CFE group (L-CFE)	10 U/mL CFE + 2 μmol/L PHZ + Embryo culture water
Medium-dose CFE group (M-CFE)	20 U/mL CFE + 2 μmol/L PHZ + Embryo culture water
High-dose CFE group (H-CFE)	30 U/mL CFE + 2 μmol/L PHZ + Embryo culture water

**Table 2:** Effects of CFE on the total distance traveled by zebrafish.

Groups	Total Travel Distance (mm)
Control group	4244.07 ± 182.72 ^a^
Thrombus model group	1117.90 ± 195.30 ^e^
Positive control group	2877.69 ± 208.27 ^c^
Low-dose CFE group	3615.78 ± 189.54 ^b^
Medium-dose CFE group	2623.98 ± 204.28 ^c^
High-dose CFE group	1903.14 ± 218.36 ^d^

Note: Different lowercase letters indicated statistically significant differences between groups (*p* < 0.05) as determined by multiple comparison tests.

**Table 3:** Effects of CFE on the average distance moved by the zebrafish.

Groups	Average Moving Speed (mm/s)
Control group	14.20 ± 0.65 ^a^
Thrombus model group	3.73 ± 0.42 ^f^
Positive control group	10.28 ± 0.51 ^c^
Low-dose CFE group	12.06 ± 0.37 ^b^
Medium-dose CFE group	8.99 ± 0.43 ^d^
High-dose CFE group	5.88 ± 0.55 ^e^

Note: Different lowercase letters indicated statistically significant differences between groups (*p* < 0.05) as determined by multiple comparison tests.

## Data Availability

The original contributions presented in this study are included in the article. Further inquiries can be directed to the corresponding authors.
